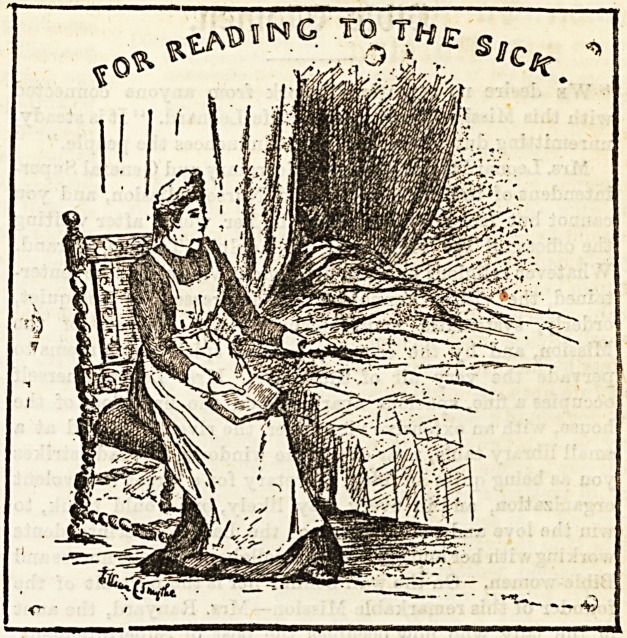# The Hospital Nursing Supplement

**Published:** 1891-09-05

**Authors:** 


### "The Hospital" Nursing Mirror.

Being the Extra Nursing Supplement of "The Hospital" Newspaper.

Contributions for this Supplement should be addressed to the Editor, The Hospital, 140, Strand, London, W.C., and should have the word "Nursing" plainly written in left-hand top corner of the envelope.

### En Passant.

THE LIVERPOOL NURSES.—The patient at the Liverpool Infirmary who was so anxious to do justice to the kindness of the nurses has brought a hornets' nest about their heads, and a hot war of words is raging in the Liverpool papers on the subject. Not all of us can bear to hear our fellow creatures praised. This seems to be the case with the husband of a woman who was a patient at the Infirmary, and who, he alleges, received very sorry treatment there. He is very anxious to bring the details of the matter before the public that they may have a true view of the institution nursing.

THE NURSES' CO-OPERATION.—A correspondent who has lately visited the institution writes: "In February I went to the modest little opening of the Nurses' Co-operation, and yesterday, anxious to know how the scheme had fared, I again found myself in the cosy sanctum which, in spite of the business done there, is eminently attractive as a resting-place for others besides weary nurses. The scheme has answered beyond the wildest dreams of its promoters, who consisted of a little band of nurses who saw no reason why they should not enjoy the full benefit of their own earnings, which usually averaged two guineas a week, which sum was paid to the Nursing Association to which they belonged, while they were engaged by the Association at a modest salary of, say, £25 a year. The nurses, who must possess first-rate qualifications, or they will not be enrolled as members, get engagements through the Co-operation Society, and take the whole of their earnings less 7½ per cent., or 3s. in every two guineas, which is handed over to carry on the working expenses of the society. Should this 7½ per cent. yield a surplus at the end of the year it will be divided among the nurses, and so great is the demand for their services that there seems a brilliant future before them."

THE COVENTRY AND WARWICK HOSPITAL.—Unpleasant rumours having been in circulation as to the alleged mistreatment of a case at the Coventry and Warwickshire Hospital. On Wednesday evening the Committee met at the hospital to consider the question. The charge, made by William Gould, working at Messrs. Bayliss, Thomas, and Co.'s cycle manufacturers, was that he broke his arm on a polishing bob, and went to the hospital to have it set by the acting house surgeon, Dr. Parsons. The bone was set, but not feeling satisfied with the treatment he went to the Queen's Hospital, Birmingham, where the arm was attended to by Dr. Sadler, one of the house surgeons. Upon returning to Coventry he made a statement to the effect that the Birmingham doctor had said the arm was set badly, that the splints were not put on properly, and that the splints were taken off and handed to others present and criticised as a specimen of Coventry hospital work. Mr. W. R. Thomas, one of the Hospital Saturday Committee, to whom the charge was made, wrote to Dr. Clay for a "written corroboration," and in Dr. Clay's absence Dr. Sadler replied "that the fracture had been well treated, that the man Gould had no reasons to offer for his complaint of maltreatment," and "that Gould was insolent and ill-behaved in the registration office." The same day, August 2nd, Dr. Sadler wrote to Dr. Parsons stating that "they put him in possession of the particulars with the idea of helping him to put down such unscrupulous grumblers, who were unfortunately too common." The Executive said Dr. Parsons was entirely exonerated from blame, but they were determined to sift the matter to the bottom in the interests of the hospital. The meeting was ultimately adjourned, and Mr. Gould is to be asked to meet the Committee.

FOR THE ADULTS.—Already a parcel of ten pairs of socks has reached us from E. W. B. towards the Christmas gifts which we hope to distribute for adult patients. Also a reader writes "I am not a nurse, so cannot enter for the sixth competition; but nevertheless I am going to send you a dressing-gown to be given away to some poor convalescent." Will all our readers remember that any gifts will be useful whether sent in competition or not?

THE NURSE AND THE MEDICINE BOTTLE.—All nurses must be interested in the verdict which Sister Collecta, of the Sisters' College, Lisborn, will receive for having administered oxalic acid to a pupil instead of magnesia, and thereby causing her death. Our hospitals have so far been singularly free from mishaps of this kind on the part of the nursing staff. Several cases have occurred lately in which the chemist has been at fault. The only subject for surprise is that mistakes do not occur oftener where the dispensing is left, as it frequently is, to a young assistant. We expect more care on the part of a nurse, but in Sister Collecta's case we have yet to hear if there were not circumstances in extenuation.

NURSING ASSOCIATION FOR WAPLINGTON.—On the 13th ult., in response to an invitation from Lady Margaret Bickersteth, a large and influential company consisting of ladies and gentlemen of the neighbourhood, assembled in the drawing-room, Waplington Manor, to consider a scheme for providing trained nurses for the rural districts. The Right Hon. Lord Herries presided. The Chairman proposed the first resolution—"That an Association be formed for the purpose of providing nurses for the sick in country districts, and that it be called the Wilton Beacon Benefit Nursing Association." He suggested that there should be a union of parishes in a 6 or 3 miles radius, and a certain number of nurses be provided, who would not be above doing even menial work as well as attend to the wants of the sick. It was decided that the expense be met by contribution of £2 per 100 inhabitants. Dr. Wright seconded the resolution and give his experience as a docter of the bad results of inefficient nursing, and spoke highly of the York Hospital nurses and other nursing sisters. It was decided that one nurse would be sufficient for 1,400 people, and that the cost of each nurse would be £28 per year. That subscribers would have her services at a reduced charge. It was to be understood that it was not a charity but a benefit society, and that a minimum subscription of 2s. per year must be paid, as it was not intended to give the services of the nurses gratuitously, but by combination to get better value and at a reduced charge. Mr. Wilson said that all the labourers should be encouraged to become subscribers. On the termination of the proceedings, the ladies remained to discuss details with Lady Margaret, and make preliminary arrangements.

### Lectures on Surgical Ward Work and Nursing.

By ALEXANDER MILES, M.D. (Edin.), F.R.C.S.E.

### XXXIV.—SURGICAL INSTRUMENTS.

I PROPOSE in the next few papers to consider the subject of surgical instruments, describing and indicating the uses of the more important of these. As far as is possible the description should be read with the instruments before you, as it is only by this means that you can obtain any useful knowledge of them, and, of course, it is needless to say, that where practicable every instrument should be applied to its appropriate use. Space does not permit that I should describe in detail the instruments used in each surgical operation, and I do not attempt here any systematic classification. As a rough guide, however, I shall follow the steps of an ordinary amputation, say, through the thigh, and describe the various instruments suggested at each step.

### INSTRUMENTS USED IN AN ORDINARY AMPUTATION.

I. TOURNIQUETS.—(a) *Esmarch's Tourniquet for the bloodless operation* consists of two pieces—a strong elastic bandage and a thick piece of elastic tubing, fitted at one end with a few chain links, and at the other with a hook, by means of which it is secured. The elastic bandage is applied tightly to the limb, passing from below upwards, and so driving all the blood out of the vessels. At the upper limit, just above the seat of amputation, the powerful tubing is fixed, and so prevents the entrance of blood into the part on the removal of the bandage. The tubing should

PETIT'S SCREW TOURNIQUET.

be applied over a few turns of *wet* cotton bandage. This prevents it from injuring the skin, and from slipping. By this means the part to be removed is rendered absolutely exsanguine, and not only does the patient lose no blood at the operation, but after it he has proportionately more blood in his body than he had before. The disadvantage of this instrument is that it cannot be slackened gradually to ascertain if all vessels have been tied as with Petit's Screw Tourniquet. It must, in fact, "be either off or on." *(b) Foulis' Elastic Tourniquet* consists of a piece of strong indiarubber tubing about two feet long, and furnished with a simple catch. The tube is stretched and passed once or twice round the limb and then fixed into the catch. In applying this instrument the limb should be elevated for a few minutes so as to allow it to become devascularised, and then a turn or two of moist bandage should be *loosely* rolled on, and over this the tourniquet is to be rapidly and tightly applied. It is important to put on the tourniquet quickly and firmly, because by so doing you cut off all the blood going into the limb as well as the venous return, and so attain the object of a tourniquet, whereas if this point be neglected the venous current is stopped while the more forcible arterial flow goes on, and the tourniquet becomes a means of engorging rather than of emptying the limb. With Foulis' tourniquet the vessels when cut do bleed a little, but this is, to some extent, an advantage, because it enables the surgeon to see and secure them before removing the tourniquet. (c) *Petit's Screw Tourniquet* is more complicated in construction than the others, but equally simple in principle. It consists of a metal frame, of two plates perforated by a screw, and threaded through these is a strong inelastic belt fitted with buckles, and a pad to go over the main artery of the limb. One or two points must be attended to in fitting up this instrument. (1) Make sure that the band is properly threaded into the brass plates. To insure this it must pass twice through each outer division in the under plate, and not at all through the inner division. If properly threaded no brass is visible on the under surface of the instrument, while if wrongly done the inner bar on each side is seen. (2) Be careful before beginning to thread the tourniquet that the buckle is turned so that it will catch when placed on the limb. (3) Approximate the two plates before beginning to apply the tourniquet. This tourniquet is also applied over a turn or two of moist bandage, and the pad is placed over the main blood vessel of the limb, so that when the screw is brought into action the blood supply will be cut off as efficiently as possible.

The great advantage of Petit's instrument over most others is that it may be slackened gradually, and so any vessels which have escaped the surgeon may be seen, the screw tightened again, and these tied without undue loss of blood to the patient. (d) *Elastic Webbing,* of any sort may be used as a tourniquet, being put one layer over the other. The point of importance is to make the first turns rapidly and very tight.

*(To be continued.)*

### A Few Words to Nurses.

OF all the wonderful changes to which this present century has given birth, there have been none more valuable than the movement, which has improved the old style of nurses from off the face of the earth, and substituted for them the trained, capable, and energetic class of women who have taken their place.

As any man who was incapacitated from gaining his living in other ways, was in old times considered good enough to be a schoolmaster, so it would appear, that any woman too old or too idle for ordinary occupations, was free to prey upon her neighbours in the capacity of a nurse.

Not that all were so bad as Mesdames Gamp and Prig; most of them were simply incompetent, while there were here and there a few bright examples of upright women who, with good common sense, and an aptness for learning from experience, brought comfort into both the sick-room and the nursery, and remained honest, faithful friends till death severed the tie. But these were exceptions, even in private life, while there is no doubt that nothing much too bad could be said of the wretched beings to whose care the sick in the public hospitals were relegated.

The novelist, however, with his humorous pen, opened the eyes of the public to the abuses prevalent in their midst; while the increased knowledge of the medical profession and the great skill of the modern surgeon necessitated adequate coadjutors, or their patients would die when they passed from the operators' hands. The ball thus set rolling has gained as it went on in size and strength, until nurses have become a mighty power in society. The readers of THE HOSPITAL have followed the whole movement step by step, and it is needless, therefore, to descant further on the subject; but its very success may have originated dangers which it would be well to guard against.

Gratitude and sympathy are alike called out in behalf of the nurse, whose life is a most trying and arduous one, but recalling the old-world saw of Lindley Murray, "Flattered and applauded, he became vain"; may not the fêting and patting on the back which the profession has received during the last two years tend to turn the heads of the least well-balanced members? Nurses have their own peculiar joys and sorrows; they are but women after all, though they often come to us as blessings, we were about to say angels in disguise, yet they should not forget that there are two sides to every question, and that even patients and their relatives may not unreasonably expect some sympathy and consideration.

The nurse has to guard against her own human weaknesses. Many a tough battle with impatience or ill-temper has been fought out silently by the sick bed, and in the hospital ward, amidst the toil and anxiety of everyday life. The aims and motives of those who join the nursing ranks are various. A wish for change, a desire to escape the monotony of home life, incite the choice of some, while a natural taste for nursing, or the necessity of gaining a living, induce others to take up the work.

These motives, harmless and even praiseworthy, are not sufficient to carry us triumphantly through the heat and burden of the day. It requires a surer foundation on which to build, and help from a higher power than our own weak wills, to make us tread patiently in the footsteps of our "Great elder Brother."

A few words of counsel and advice from one who sympathises, heart and soul, with both nurse and patient, may not be unacceptable, now and then, to those who have given themselves to this special following of Him who "Himself took our infirmities and bare our sicknesses."

### The Princess of Wales and the Nurses.

Photographs are pouring in for the screen which the First and Second Thousand nurses have decided to offer for the acceptance of the Princess of Wales on her next birthday. As not more than 1,000 photographs can be placed on the screen those nurses who wish to show their appreciation of the Princess's kindness to them should send carte and postal order for 2s. immediately, to Screen, care of Miss Pritchard, The Lodge, Porchester Square, London, W., or they may be too late. Postal orders from the following Nurses are acknowledged: Nurse Waters, postal order for 20s.; Collins, Tridson, M. A. Wilson, E. Marshall, F. G. Davis, Osborne, A. L. Middleton, M. Hayward, A. Hayden, M. E. Turner, A. J. Byrne, K. E. Stannett, M. Moulton, M. Thomas, W. A. Fitchett, F. T. Westmore, M. A. Parlett, A. L. Phillips, M. A. Watson, B. Berry, Tatt, M. A. Mitchell, H. M. Hill, E. Boul, H. Child, E. M. Allen, S. E. Starling. E. A. Steer, T. Keogh, A. E. Bunster, S. A. Elmer, A. Wallen, E. Lewis, J. M. Gilpin, L. M. Dunkley, M. M. Dobbs, H. L. Arnold, M. A. Mackie, A. Botwood, A. Badder, Readings, R. Cumberland, H. Horton, L. Jays, S L. Peter, E. Farrington, A. Fletsrof, I. Pringle, E. H. Windeler, C. Stewart, E. Cotton, A. Read, E. H. Woomack, C. R., Attfield, E. Jarman, E. Barfield, H. E. Andrews, H. W. Russel, S. A. Mallinson, M. Basham, E. E. Poole, E. Hutchinson, J. Parker, E. Meadows, M. A. Plowman, M. Hill, E. Clarke, Payn, S. J. O'Leary, C. J. Featon, A. Soper, A. Bryan, A. R. Webster, H. Wright, M. A. Crisp, R. G. Jackson, A. Martin, L. A. Smith, M. M. J. Hawthorne, K. Pitchford, O. Nicholas, Freakson, and E. Stone.

Up to the present time 330 photographs have been received and 273 have been promised. The screen will only contain altogether 750 photographs, so that there are still required 147. Any nurse who desires to join in this presentation must therefore send her photograph with a postal order for 2s. to "Screen," Miss Pritchard, The Lodge, Porchester Square, W., not later than September 15. It is hoped that the full number will be completed by that date, and that those nurses who are really grateful to the Princess of Wales for all she has done for them will hurry up. It is found that the screen will cost more than was anticipated, so that any Sister, Matron, or nurse, who is willing to contribute a sum not exceeding 5s. is invited to do so should she be so disposed. These further contributions should also reach Miss Pritchard by the 15th inst. Full particulars of the arrangements for the presentation will be published about the second week in November.

### TO CONVALESCENTS.

We have been very ill, we have had a hard time of it, but now, thank God, the tide has turned and health is flowing into our veins. A great writer says: "Convalescence is like the renewal of youth, or like a bright day in October cheering the gloom of the falling year." Alas! it soon passes away, and we shall have so many restless, weary days before our health is re-established that it may be as well to see how we can best turn to account the passing hours. One day we seem strong and hopeful, the next feeble and pining, but if we try to make the best of these ups and downs, bearing the latter patiently and rejoicing in the former, we shall get well all the sooner. I knew a friend who some years ago fell into a chronic state of invalidism after a severe attack of rheumatic fever. She was told by her doctor she might live for years, but he held out no hope that she would ever be much better or able to walk again. At first she was much depressed, it seemed such a death in life for her, but being naturally of a cheerful disposition she determined to make the best of things and count up the mercies which remained to her. She took herself to task and said, here have I a comfortable home with food and clothing and many kind friends who sympathise with me. If I always repine and complain of my ailments they will vote me a grumbler, and soon leave off visiting me, so I must keep down impatience, fretfulness, and discontent, and have a grateful heart to my heavenly Father, who, in taking away the blessings of health and strength, has left my mind clear and the use of my sight. So she possessed her soul in patience, and slowly, almost imperceptibly, she crept back to life. It was a hard task she had set herself, but she did not attempt it in her own strength, she prayed to Him, who, when on earth, healed the sick and made the lame to walk, to give her back such an amount of health as He should think good for her, and after some years of patient waiting she again walked and took her place in her family. I think such a pause in life must have been given her for a set purpose. She had had a busy life with much responsibility, and this gave her a tendency to think nobody, hardly "Providence, could get on without her," but the world has never yet stood still for the want of one man, and was not likely to do so now for the lack of her, she learnt to think more humbly of herself and to become reconciled to see the race of life pass by her. Let us hope her trials also taught her that the remainder of her days was not her own, but had only been lent to be devoted to God's glory and service.

What one weak woman could do can be done by others. You may be young and have a good constitution, and will regain health rapidly, but do not forget that you, too, have been into the jaws of death, and were rescued. Then keep a grateful heart to Him who has given you more time for a due preparation for Eternity.

### Bible Women.

"We desire no sensational work from anyone connected with this Mission," writes Mrs. Selfe Leonard. "It is steady, unremitting duty-doing work that influences the people."

Mrs. Leonard is the Honorary Secretary and General Superintendent of the Bible-women and Nurses' Mission, and you cannot but feel a twofold force in her words after visiting the offices of the Mission in 2, Adelphi Terrace, Strand. Whatever religious or even irreligious views may be entertained, the visitor cannot but be impressed by the quiet, orderly, systematic management of the business of the Mission, and by the earnest, fervent spirit which seems to pervade the very air of the place. Mrs. Leonard herself occupies a fine, spacious apartment on the first floor of the house, with an extensive view over the river. Seated at a small library table, near one of the windows, this lady strikes you as being quite an ideal Secretary for a great benevolent organization, and in every way likely, one would think, to win the love and respect both of the Lady Superintendents working with her and her humbler collaborators, the nurses and Bible-women. On the wall behind her is the portrait of the founder of this remarkable Mission—Mrs. Ranyard, the aunt of the lady who now occupies the post of Superintendent. The very type of a motherly face is that that beams down upon this large council-room, shrewd, strong, penetrating, but, above everything, good and kind. Four or five-and-thirty years ago the tender heart displayed in this benevolent face was touched by the terrible condition of the poor of Seven Dials, and she set a Bible-woman to work among them—a real, genuine missionary—and, of course, the two good people soon found that Bibles were not the only things needed in the Seven Dials, and, says her biographer, "They sold for a halfpenny a printed recipe for a nourishing soup that could be made for sixpence, and they lent a saucepan to make it in. They started a clothing club and a sewing meeting; they thought and planned for individual cases in all sorts of ways. Thus, little by little, there grew up a domestic as well as a Bible Mission."

"And what number have you now in your ranks, Mrs. Leonard?" inquired a visitor.

"We have 140 Bible-women and 75 nurses, whose duties are *absolutely* distinct from those of the Bible-women."

"And from what class do you select them?"

"They are all working women—women who belong to the people amongst whom they labour—for we believe for poor people's homes women of the working class are far the best. They must know more about the people and their trials and difficulties than ladies can possibly do, and we are sure that the poor feel more at ease with them."

"How do you get them?"

"They come to us. Young women get to know our Mission from our nurses and others, of course, and we have a good many applicants on our list."

"And how do you select them?"

"We investigate the characters and antecedents of candidates very carefully. They must be women of thoroughly good Christian character. We make a great point of this. If we are satisfied as to this, and find reason to believe that they are naturally adapted to nursing work, we give them a a year's training, partly in one of the general hospitals, partly in a lying-in hospital, and then a practical training in one of our districts, where, I need not say, they find the work of nursing something very different from their experience of it in hospital wards."

"And at the end of her year of training will she have a specified district?"

"Yes. You see we have a large part of the poorest portions of London mapped out," and as Mrs. Leonard spoke she took up a large volume, on each page of which was a section of the map of London, very precisely marked out in districts. "You see," continued the Superintendent, "we have our women all over the metropolis. We are the largest association of the kind in London."

"But do these women work in these districts without any oversight?"

"Oh, no; we have 160 Lady Superintendents who spend some time each week in the inspection of their books and in advising and instructing the nurses and Bible-women. Then we also have nurse pioneers. They were originally just what their name implies—those who went first on the district and prepared the way for Bible-women and nurses. Now, however, their work is somewhat different. They are women of great experience in district work, and inspect the work of the nurses, visiting every district in turn, and reporting to the Mother House."

"And do your people wear uniforms?"

"Yes; they have plain but suitable uniform for the work. It is just a plain dress and a cloak, and a linen apron and collar, and a neat bonnet with strings."

"The cost of their preliminary training must be considerable. What hold have you on them afterwards?"

"We require them to enter into an engagement for a term of three years from the expiration of their training."

"And their pay?"

"They get fifteen shillings a-week at the outset. We reckon that every nurse costs the mission in one way and another—salary and uniform, and supplies of necessaries and medical comforts—from £55 to £60 a-year."

"Your people do not go empty-handed, then?"

"Oh, dear, no; every nurse carries a bag supplied with bandage, ointment, lint, a macintosh sheet, flannel and towels, and many other things, such as a medicine glass, a clinical thermometer, forceps, scissors, a feeding cup, a syringe, and tin cans for soup and lotion and oil. We often find, also, that nourishing food is really more necessary than medicine, and in such cases we supply what is required."

"And how is all this maintained?"

"We get local subscriptions sufficient to make most of our districts self-supporting. We have a central fund from which we assist new districts for a time, and there are some localities so poor that we get little or nothing locally. In such cases we carry on the work entirely by the Nurse Fund of the Mission."

Further conversation elicits the fact that this unobtrusive but eminently practical mission has developed various agencies in connection with it, such as render it one of the most complete and efficient agencies for usefulness among the poor it is possible to conceive. Eight or nine years ago they opened at Southend a small house as a Convalescent Home for Women. Of course this proved immensely valuable in such a work as theirs, and subsequently three very nice villas were presented to the Mission for an extension of this admirable branch of the nursing benevolence; and thus in a single year, 108 men, 294 women, and 52 babies, most of whom had never been to the seaside before, were enabled to get rest and change and fresh air, and a brief, but no doubt impressive, experience of something like real home life—clean and sweet and cheerful, and free from the gloom of daily anxieties and worries. How much even the briefest experience of the kind may do, not only to make hearts braver and stronger for the battle of life, but to awaken thought and kindle higher hopes and aims, can be only very imperfectly realised. Then they have a dormitory for needlewomen and servants out of place in the vicinity of Drury Lane; and a Home of Rest, that surely must be at times sadly needed, for their own Mission Women, at Brighton.

This bright, pleasant, sunshiny house on Adelphi Terrace seems, in fact, to be the centre of a great circle of active philanthropy of the most wholesome and vivifying character. There are some of us perhaps, who, if we begun to discuss theology with some of their Bible-women, might be disposed to fall out a little with them, but none of us ever fall out with the practical work of nursing the sick and sympathising with the sad and suffering. It is a splendid work, most admirably organised and carried on in the noblest and purest spirit of benevolence, and it is as unobtrusive as it is good and useful. The modesty of the whole Mission seems, indeed, to be characteristic of those who support it. It was merely "a valued friend" who took the first house for a Convalescent Home at Southend, and it was merely "another friend" who extended the work. A very remarkable anonymous friend mysteriously appeared in 1861, and, after close inquiry, left a donation of £200. Next year he came again, went carefully into every detail of the work, and left a larger sum. Again and again he came, always increasing his donation and always stipulating that no attempt should be made to discover his name or fund his money. For many years latterly this unknown benefactor has contributed £3,000 a-year to this Mission, and altogether he gave £60,000. His sudden death during the epidemic of influenza has been a sad loss to the mission; but if those who have money to dispose of for charitable purposes could only know the amount of service these good women are doing in the slums and dark places of London, three thousand a-year would soon be made up.

### Presentations.

MISS GOLDMAN, who has occupied the position of Sister of the female wards of the General Hospital, Launceston, Tasmania, has unfortunately been obliged to resign through ill-health. During the year Miss Goldman spent in the hospital she made many friends, and her enforced resignation is a matter of deep regret to those officially connected with her. As her illness will necessitate a long rest from work, many friends showed their sympathy in a most substantial manner by presenting her with a cheque to help to defray the expense to a warmer climate. Miss Goldman was trained at the Royal Infirmary, Bristol, where she was nurse three and a-half years, she was afterwards engaged in private nursing at home and abroad, and we are sure her many friends will be sorry to hear of her ill-health.

ANCOATS HOSPITAL.—Sister Sidebottom was presented, upon the occasion of her marriage, by the Matron and nurses with a case containing five o'clock tea service; a set of silver spoons from the resident doctors, brass spirit kettle from the President of Ladies' Committee, besides other tokens of the esteem in which she was held by various friends of the hospital. Sister Pumphrey received, upon her resignation, two volumes of Quain's Dictionary and illuminated address from the honorary staff. Miss Radford succeeds as senior Sister, and Miss Taylor promoted to be Sister in children's ward.

### Notes and Queries.

### Queries.

(39) *New Zealand.*—What openings in mental nursing are there in New Zealand? Are there any hospitals for the insane?

(40) *A Reader* writes:—Would you oblige me by sending particulars of any convalescent homes that would take my father? He is suffering from lung disease, and the doctor has ordered him a change into a drier air. He could afford to pay a small sum per week.

(41) Can you tell me of an institution where a woman, whose friends could pay only a very small weekly sum, can be treated for hysteria?—*Nurse R.*

### Answers.

*Emmeline.*—You cannot do better than get Miss Catherine Wood's book.

*Newsagent.*—Articles of the "Diseases of the Fingers" are now appearing in our pages. See "The Practitioner's Mirror" for the last month.

*A Constant Reader.*—Get "A Manual of Testing," by K. M. Heanley. Published by Churchill; price (we believe) 1s. 6d.

(35) The Little Sisters of the Poor have a home for the aged close to Vauxhall Station; it is open to all denominations.

(36) Get your shoes from Parker's, 145, Oxford Street.—*J. Wilson.*

(34) A letter, dated July 6th, from Dunedin, says, "The weather is bright, cold, and dry. Water is being sold by the bucket, as the rains still delay."

*Nurse Lucy.*—See answers this week and last to (34), also see query (39).

### Everybody's Opinion.

[*Correspondence on all subjects is invited, but we cannot in any way be responsible for the opinions expressed by our correspondents. No communications can be entertained if the name and address of the correspondent is not given, or unless one side of the paper only be written on.*]

### OUT-DOOR UNIFORMS.

A "TEN YEARS' NURSE" writes: We nurses, in our seaside town, feel we are quite out of touch with the London nursing world, and do not know what is considered sufficient out-door uniform in the metropolis. We had always thought aprons formed part of the in-door uniform of nurses, but such is evidently not the case, for several nurses who are down here with patients walk beside their bath chairs with cloaks flowing open from the neck, showing, sad to say, very often dirty aprons beneath. Not only do nurses appear on the parade in their aprons, but the other Sunday I was coming out of church, and saw a nurse entering, I suppose, for the second Celebration, in a very dirty apron; one that I should have been ashamed to appear before a patient in, much more, therefore, to have gone to God's house in. For my own part, I object to out-door uniforms, as they not only attract notice to the nurse more than is altogether pleasant, but it must often be very objectionable to an invalid who is sensitive about appearing as such. When a dirty apron and dirty bonnet-strings are the most conspicuous part of a uniform, then I think it is time to draw the line. It does not seem to me that the dye from the cloaks can be altogether antiseptic, therefore cannot be quite the right thing for an apron that is worn in a sick-room to be brought into such close contact with it.

### Appointments.

[It is requested that successful candidates will send a copy of their applications and testimonials, with date of election, to THE EDITOR, The Lodge, Porchester Square, W.]

COLERAINE COTTAGE HOSPITAL.—Miss M'Donnell, daughter of Dr. M'Donnell of Dublin, is giving her services as Nurse Superintendent of this new hospital.

RADCLIFFE INFIRMARY.—Miss F. Masson of St. Thomas's has been appointed Matron at Oxford in place of Miss Lucas, who resigns after six years' excellent work.

PHILLIPS MEMORIAL HOSPITAL, BROMLEY.—Miss Ellen Hyde has been appointed Matron in place of Miss Davey. Miss Hyde has worked at Tunbridge Wells, and as Sister at Worcester Infirmary; last winter she took temporary duty at the hospital to which she is now appointed.

DERBYSHIRE ROYAL INFIRMARY.—Miss Bagnall-Oakeley has been appointed Matron of this infirmary; she trained at Charing Cross, and held the post of Sister there, afterwards acting as Matron at Wolverhampton and the West Hertshire Infirmary. Miss Bagnall-Oakeley holds excellent testimonials, certificates for nursing and dispensing, and the diploma of the L.O.S.

QUEEN VICTORIA'S JUBILEE INSTITUTE FOR NURSES.—The Queen has approved of the following additional names being entered on the roll of Queen's Nurses for Nursing the Sick Poor in their own Homes:—Superintendents—Katherine Persse, Paddington; Jane Wade, Chelsea; Louisa Taylor and Helen Sargent, East London; Ruth Wood, Glasgow. Nurses—Annie Julia Franklin and Ella Mason Morgan, Chelsea; Alice Mary Wallick, Woolwich; Alice Maud Pepper, Paddington; Alfrida Lindolm, Hampstead; Isabella Cairnie, Londonderry; Catherine E. Jones, Conway; Emily Reedee, Wolverhampton; Caroline Ann Blackmore, Stamford; Susan Reid, Rugby; Mary J. Murphy, Hertford and Bengeo; Isabella Andrews, Isabella Armstrong, Mary Armstrong, Eliza Fraser, Annie Ford, and Ellen Wilcox, Edinburgh; Mary Monckhouse and Margaret Tattam, Kilmarnock; Jessie Allen, Alice Maria Epps, Frances Homer Hunt, Mary Elizabeth Neill, Elizabeth Sutherland, Annie Towers, and Elizabeth Sinclair White, Glasgow; Margaret St. Clair, Christina Watson, Annie Emily Homan, Fanny Frances Howell, Louisa Charlotte Grace Yeats, and Jane Alice Berry, Dublin; Eliza Blackburne, Londonderry.

### A Little Leader.

"A little child shall lead them."

It was a Sunday afternoon in April. Through the tall windows of the surgical ward the bright spring sunshine looked in on the white beds, and brightened with its smile the wan faces there. A little cheerful stir was apparent, for it was a visiting day. Though not a children's hospital, there were always some little ones in this ward, making an extra call on the care and patience of the nurses, but, in spite of that, I really think they loved the babies best.

In one of the beds lay a tiny child, apparently only six or eight months old, but in reality he was three and a half years. His patient little face, with large pathetic eyes, long lashed, and wide blue-veined brow, scarcely shaded by the clustering rings of thin golden hair, told something of the suffering of his short life.

Infinite mystery of pain! and never more inexplicable than when it seems the birthright of an innocent child, born only to suffer and die!

Seven weeks before, little Arthur had been brought to the hospital, to be treated for bone disease in the leg, which, after several operations, had been amputated entirely. He had rallied wonderfully for a few days, but was now growing daily weaker.

There he lay eagerly awaiting his father's weekly visit, his eyes fixed on the distant doorway, hugging closely his one treasure, a woolly toy sheep—his little hot hand clasping a crumpled scrap of paper. This was a letter to mother, which he was to put in daddy's pocket. He had a fondness for scribbling, and the kind little "Pro" who was on duty that afternoon, had given him a dainty gold-edged leaf from her private note book for this purpose, knowing that this little message from her dying child would speak to the invalid mother's heart.

"Daddy not come," he said in a pitiful voice, as the nurse approached; the tears were very near, but kneeling down beside him, very gently did she tend and soothe him, whispering of the Good Shepherd who was coming soon to take him to a beautiful place where he would never be tired any more, nor in pain, but would run about and play with other happy little children, and gather flowers his "very own self." "Kind Man!" said the child, smiling at the picture which nurse had hung at the foot of his cot, and stroking the soft fleece of his toy. "Kind Man! Not hurt the wee lamb!"

But, soon, a faint cry of welcome announced that Daddy had come.

He's far, far through to-day, nurse," said Tom Forster, half-an-hour later, when she came round again. A look of assent and a gentle sigh was the only answer, as Nurse stood looking down on the wasted little face, which, now that the flush of excitement had faded, showed wan and weary in the afternoon light. "His mother is very weak to-day, I could scarce leave her, but she would not have the bairn disappointed."

The minutes passed. The child rested peacefully. Suddenly he opened his eyes, "Want mother," he said, with a little gasp. "Want mother," then smiling as his eyes rested on the picture, and making a little gesture with his hand toward it, "Kind——Man," he whispered, and, as his eyes closed, "Daddy——come——too!" A look passed between the two watchers, but no word was spoken, none was needed, both knew that the child was in heaven!

"How shall I tell her?" said Tom Forster, as he walked home, his mind possessed with a strange sort of feeling that somehow all would be well. And it was! He entered his desolate home only to find that another messenger had been there.

He bent to kiss the waxen brow of his dead wife, while like an angel's whisper the faint little voice sounded in his ear, "Daddy—come—too!" He placed the baby's letter on her breast, and sunk on his knees.

### "A 'HARBOUR OF REST' IN THE LAND OF OLD AGE."

The Royal National Pension Fund for Nurses.

Fair nurses of Britain, the harbour awaits you!
Spread your sails to the wind while it's gentle and fair;
Heave ahead; "Cast the lead," if doubt should beset you!
No shallows or shoals found, whilst journeying there.

Beacons to guide you—Royal Prince and Princess—
Reliable charts, by reliable men,
Experienced pilots—but I'll "belay" the excesses
Of lauding known merits again and again!

Weigh anchor! Don't tarry! lade ye your freights there!
Plain sailing to port o'er life's troubled sea.
Buoys mark the Channel! Perish all doubts there!
"Unprovided old age" leave away on your lee.

With one hearty "God bless you" to founder and compeers,
Launch away on the voyage made easy and plain,
Set a course for old age! You know now how "land bears"
Where snug berths in the "Habour of Rest" await claim!

W. B. Hudson.

### Amusements and Relaxation.

SPECIAL NOTICE TO CORRESPONDENTS.

**Third Quarterly Word Competition, commenced July 4th, 1891, ends September 26th, 1891.**

Competitors can enter for *all* quarterly competitions, but no competitor can take more than *one first prize* or *two* prizes of any kind during the year.

Proper names, abbreviations, foreign words, words of less than four letters, and repetitions are barred; plurals, and past and present participles of verbs, are allowed. Nuttall's *Standard* dictionary only to be used.

N.B.—Word dissections must be sent in WEEKLY not later than the first post on Thursday to the *Prize Editor*, 140, Strand, W.C., arranged alphabetically, with correct total affixed.

The word for dissection for this, the TENTH week of the quarter, being

"PARTRIDGE."

| Names. | Aug. 27th. | Totals. | Names. | Aug. 27th. | Totals. |
|---|---|---|---|---|---|
| Paignton | 12 | 253 | Christie | — | 44 |
| Psyche | 13 | 254 | Dulcamara | 11 | 245 |
| Hope | — | 47 | Nurse J. S. | 12 | 253 |
| Lightowlers | 13 | 266 | Qu'appelle | 13 | 258 |
| Wizard | — | 179 | E. M. S. | — | 68 |
| Wyameris | — | 46 | Jenny Wren | 12 | 236 |
| Dove | — | 46 | Carpe-diem | — | 65 |
| Punch | — | 181 | Grannie | — | 36 |
| Ivanhoe | 13 | 216 | Nurse G. P. | 7 | 144 |
| Tinie | — | 93 | Goodnight | — | — |
| Agamemnon | 13 | 259 | Gamp | — | — |
| Nurse Ellen | — | 86 | Charity | — | — |

### Notice to Correspondents.

All letters referring to this page which do not arrive at **140, Strand, London, W.C.**, by the *first post on Thursdays*, and are not addressed PRIZE EDITOR, will in future be disqualified and disregarded.

N.B.—Each paper must be signed by the author with his or her real name and address. A *nom de plume* may be added if the writer does not desire to be referred to by us by his real name. In the case of all prize-winners, however, the real name and address will be published.

## Figures and Tables

**Figure f1:**
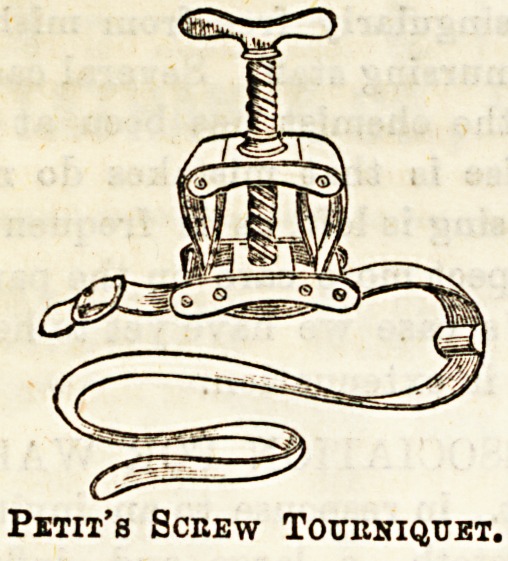


**Figure f2:**